# The “Globularization Hypothesis” of the Language-ready Brain as a Developmental Frame for Prosodic Bootstrapping Theories of Language Acquisition

**DOI:** 10.3389/fpsyg.2015.01817

**Published:** 2015-12-09

**Authors:** Aritz Irurtzun

**Affiliations:** CNRS, IKER (UMR 5478)Bayonne, France

**Keywords:** globularization, prosodic bootstrapping, language development, language acquisition, postnatal development

## Abstract

In recent research (Boeckx and Benítez-Burraco, 2014a,b) have advanced the hypothesis that our species-specific language-ready brain should be understood as the outcome of developmental changes that occurred in our species after the split from Neanderthals-Denisovans, which resulted in a more globular braincase configuration in comparison to our closest relatives, who had elongated endocasts. According to these authors, the development of a globular brain is an essential ingredient for the language faculty and in particular, it is the centrality occupied by the thalamus in a globular brain that allows its modulatory or regulatory role, essential for syntactico-semantic computations. Their hypothesis is that the syntactico-semantic capacities arise in humans as a consequence of a process of globularization, which significantly takes place postnatally (cf. Neubauer et al., 2010). In this paper, I show that Boeckx and Benítez-Burraco's hypothesis makes an interesting developmental prediction regarding the path of language acquisition: it teases apart the onset of phonological acquisition and the onset of syntactic acquisition (the latter starting significantly later, after globularization). I argue that this hypothesis provides a developmental rationale for the prosodic bootstrapping hypothesis of language acquisition (cf. *i.a*. Gleitman and Wanner, 1982; Mehler et al., 1988, *et seq*.; Gervain and Werker, 2013), which claim that prosodic cues are employed for syntactic parsing. The literature converges in the observation that a large amount of such prosodic cues (in particular, rhythmic cues) are already acquired *before* the completion of the globularization phase, which paves the way for the premises of the prosodic bootstrapping hypothesis, allowing babies to have a rich knowledge of the prosody of their target language *before* they can start parsing the primary linguistic data syntactically.

## 1. Introduction: the globularization hypothesis

According to a recent article in this journal by Boeckx and Benítez-Burraco (2014a), “much work in neurolinguistics has unintentionally emphasized the externalization component of language, since morpho-phonology is perhaps the easiest aspect to single out linguistic tasks, even if the word “syntax” was said to be the target of the relevant works. In so doing, work on neuroimaging biased the results toward the Broca-Wernicke model, and all too quickly attributed “syntax” to Broca's area.” In contrast, Boeckx and Benítez-Burraco (2014a,b) have advanced the hypothesis that our species-specific language-ready brain (a brain which is suited for acquiring natural languages) should be understood as the outcome of developmental changes that occurred in our species after the split from Neanderthals-Denisovans, and which resulted in a more globular braincase configuration in comparison to our closest relatives, who had elongated endocasts. They propose that even if factors like brain lateralization are important, the development of a globular brain is at the outset of our language faculty, and in particular, it is the centrality of the thalamus in a globular brain that allows its modulatory or regulatory role, essential for syntactico-semantic computations (cf. *i.a*. Wahl et al., 2008).

Inportantly, globularization takes place postnatally (cf. Lieberman et al., 2002; Neubauer et al., 2010; Gunz et al., 2012), therefore, according to Boeckx and Benítez-Burraco's hypothesis, even if innately specified, the combinatorial syntactic ability of humans is not innate *stricto sensu*, but the outcome of a postnatal developmental phase. After globularization a new brain configuration is obtained whereby the thalamus occupies a central position (and a central role). As Boeckx and Benítez-Burraco (2014a) put it, “a proper characterization of the language-ready brain that does not recognize a central role to the thalamus is unlikely to be correct, for it would miss the critical engagement of the thalamus in regulating cortical activity. By providing low-frequency oscillations capable of embedding higher-frequency oscillations across distant brain regions, the thalamus provides the crucial regulation needed to form the sort of meaningful cross-modular conceptual structures that are characteristic of language.”

In this paper I discuss the developmental dimension of Boeckx and Benítez-Burraco's hypothesis and relate it to one of the most prominent hypotheses in early language acquisition studies; the “prosodic bootstrapping hypothesis” (cf. *i.a*. Mehler et al., 1988; Christophe et al., 2003; Bernard and Gervain, 2012; Gervain and Werker, 2013; Langus and Nespor, 2013).

The argument is presented as follows: Section 2 gives a brief overview of the development of the ability for phonological discrimination in human infants (an essential prerequisite for the identification and acquisition of the prosodic patterns of the target language). Section 3 presents the basic tenets of the prosodic bootstrapping hypothesis (a hypothesis that claims that language-acquiring children use prosody as a guide for inferring the basic syntactic pattern of their target language). Last, Section 4 argues for a natural combination of the globularization hypothesis and the prosodic bootstrapping hypothesis. In a nutshell, the globularization hypothesis proposes that the ability for syntactic computations is not innate, but that it rather develops *after* the postnatal globularization phase. In contrast, as the studies of early phonological development show, babies a few moths old have already a rich knowledge of the prosodic patterns of their target language. Therefore, and in line with the prosodic bootstrapping hypothesis, language-acquiring babies will be able to use their early-acquired prosodic knowledge as a guiding principle for inferring the syntax of their target language the moment the syntactic ability develops.

## 2. Early phonological abilities in human infants

Some essential ingredients for language acquisition are already present at birth. Since the seventies, a wide range of studies have shown infants' capacity for very early phonological parsing and discrimination (for an overview, see Panneton and Newman, 2012; Vihman, 2014). For instance, Eimas et al. (1971) found that infants as young as 1 month of age are able to discriminate the voice onset time (VOT) of synthetic stop consonants like /p/-/b/ in a manner approximating adult categorical perception. Similar results were obtained by Moffitt (1971) with 20- to 24-week-old infants in a study attesting the discrimination in place of articulation of different consonants. Given the limited exposure of newborn infants to speech, these results suggest that this categorical perception in a linguistic mode may be innate, and in the general debate on language nature vs. nurture, scholars such as J. Mehler have built upon these early capacities to argue for innatist “selectionist” theories of language learning whereby the baby “learns” her target language by “forgetting” others (cf. *i.a*. Mehler, 1974; Mehler and Dupoux, 1990).

What is more, the earliest fetal responses to auditory stimuli are reported at 19 weeks of gestation, long before the development of the fetal ear is complete (cf. Hepper and Shahidullah, 1994; Abdala and Keefe, 2012), and effects of very early auditory categorization have also been found *in utero*: a number of experiments have shown that third-trimester fetuses' auditory experience can influence their postnatal auditory preferences: newborns tend to quiet in response to their mothers' voice (and touch), Marx and Nagy (2015), and they also tend to prefer their mother's voice over other female's voice (cf. Mehler et al., 1978; DeCasper and Fifer, 1980; Fifer, 1981; Querleu et al., 1984; Spence and DeCasper, 1987; Ockleford et al., 1988; Hepper et al., 1993)^1^. Besides, as reported by DeCasper and Spence (1986), newborns also tend to be more reinforced by the audition of speech passages they heard *in utero* over passages they were not exposed to (and they can remember them for over a month; Granier-Deferre et al., 2011). Finally, Mampe et al. (2009) provide evidence that even the cry melodies of newborns of around 3 days of age are shaped in accordance with the intonational contours of the language they were exposed to prenatally (German vs. French). All this conforms evidence of a very early ability for the discrimination and memorization of complex sounds in newborn infants.

Regarding prosody and rhythm, there is ample evidence that newborns also have the ability for discrimination between inputs varying in different suprasegmental properties (see *i.a*. Morse, 1972; Olsho et al., 1982; Mehler et al., 1988; Karzon and Nicholas, 1989; Shahidullah and Hepper, 1994; Sansavini et al., 1997; Nazzi et al., 1998a,b; Carral et al., 2005). In particular, studies like Nazzi et al. (1998a) show that babies can discriminate between languages pertaining to different rhythmic classes [such as Japanese (mora-timed) or British English (stress-timed)] when exposed to low-pass filtered speech signals. The setting in this type of experiment shows that babies discriminate between rhythmic classes because by low-pass filtering (e.g., under 400 Hz) the speech signal, it gets a dramatic degradation of its phonemic content (i.e., the vast majority of its formant structure is removed), while it retains its rhythmic structure. Other studies employing this type of low-pass filtered stimuli (like Byers-Heinlein et al., 2010) provide evidence that language discrimination in neonates which were surrounded by a bilingual environment prenatally is robust, and that that language preference reflects previous listening experience (see also Gervain and Werker, 2013; Molnar et al., 2014a,b). Besides, other types of studies show that at 4 1/2 months babies tend to listen longer to speech samples that include prosodic pauses corresponding to syntactic units, as opposed to speech samples with pauses that break syntactic units (cf. Jusczyk and Nelson, 1996 and references therein).

All these results are to be framed in the fast (pre- and post-natal) development of the basic structures for sound discrimination in humans (whereby infants already possess an adult-like dedicated neuronal network for phonological processing at 3 months of age (cf. Dehaene-Lambertz and Baillet, 1998 as well as Peña et al., 2003; Dehaene-Lambertz et al., 2006 or Dubois et al., 2015)^2^.

Interestingly, however, early acoustic discrimination is not a human-specific ability, for it is also observed in a wide variety of other animals like guinea pigs (Vince, 1979), sheep (Vince et al., 1982) or chinchillas (Kuhl and Miller, 1975), and discrimination of languages of different prosodic types is also mastered by different species like cotton-top tamarins (cf. Ramus et al., 2000), or rats (cf. Toro et al., 2003).

Nonetheless, there is a growing amount of literature arguing that human infants go well beyond mere acoustic pattern-recognition and learning; evidence suggests that babies use the prosodic patterns of their target language in order to infer the syntactic structure underneath them in a sort of “reverse engineering.” That is, part of the knowledge obtained by babies from categorical perception is restricted to a specific area (say, learning of the vowel space or the consonantal inventory of the target language), but a subpart of the learning obtained with this innate capacity is more consequential: learning the tunes of the surrounding language helps the child making informed guesses about the syntactic structure of the language [this is so because the prosodic pattern of a language partially reflects the syntactic structure underneath (cf. Gussenhoven, 2004; Truckenbrodt, 2007; Selkirk, 2011)]. This is in a nutshell the proposal of the “prosodic bootstrapping hypothesis.”

## 3. The “prosodic bootstrapping” hypothesis

Prosody and rhythm are essential ingredients of natural language (cf. *i.a*. Brentari, 1999; Gussenhoven, 2004; Pfau and Quer, 2010) and a growing number of scholars argue that they have a close connection with other aspects of human cognition like musical aesthetics and computation, or our mathematical abilities (cf. i.a. Rebuschat et al., 2011; Arbib, 2013; Asano and Boeckx, 2015)^3^. Current literature converges in the idea that beyond the early ability for prosodic discrimination, “prosodic segmentation abilities emerge crosslinguistically some time around 8 months” (Nazzi et al., 2006, p. 296).

The rationale under the rapid acquisition of prosody could be seen as emerging from the combination of the following two factors:

- First, babies develop very early the necessary brain structures for adequately parsing acoustic inputs—and in particular human language inputs (see references above and Pang and Taylor, 2000, among others)—, and a growing number of works is emphasizing the natural “tuning up” between speech rhythm and endogenous oscillatory auditory cortical properties (cf. *i.a*. Drullman et al., 1994; Smith et al., 2002; Lakatos et al., 2005; Giraud et al., 2007; VanRullen and Dubois, 2011; Leong, 2012)^4^. In particular, neuronal oscillatory activity in the Theta band (3–7 Hz) is thought to track syllable patterns, whereas slower oscillations in the Delta band (1–3 Hz) track phrasal and intonational patterns (cf. Giraud and Poeppel, 2012; Peelle and Davis, 2012). Ghazanfar and Takahashi (2014) have argued that the same oscillatory cycles are present in macaques' lip smacking, suggesting that “lip smacking may have been an ancestral expression linked to vocal output to produce the original rhythmic audiovisual speech-like utterances in the human lineage” (see also Fitch, 2013; Martins and Boeckx, 2014, for discussion, as well as Theofanopoulou, 2015, for a recent evo-devo hypothesis according to which the myelination of the Corpus Callosum, brain asymmetry and globularity “are conjectured to make up the angles of a co-evolutionary triangle that gave rise to our language-ready brain”).
- Second, children are exposed to a very particular input (infant-directed speech), which has very specific linguistic and paralinguistic properties. Following the traditional view, infant-directed speech has a set of hyperarticulated features that help the child develop her linguistic capacities and acquire her language. For instance, infant-directed speech is typically associated with an exaggerated pitch, which is covered with emotional prosody to capture the child's attention (cf. *i.a*. Fernald, 1984; Cooper and Aslin, 1989; Fernald and Mazzie, 1991; Katz et al., 1996).

Actually, a recent study of the spectral amplitude modulation in the speech rhythm shows that (Australian English) infant-directed speech “exaggerates” the synchronization between syllable-rate modulations and stress-rate modulations, whereas adult-directed speech is dominated by syllable-time modulations. This is taken as evidence showing that infant-directed speech “is primarily stress-dominant, which could “tune” the infant brain toward stress-based speech segmentation—an adaptive strategy for boot-strapping early language learning” (Leong et al., 2014). Such infant-directed speech hyperarticulations are taken to help the child acquire the relevant phonological distinctions in her language (Kuhl et al., 1997; Cristia, 2013), a knowledge that is mostly acquired during the first year of life (cf. *i.a*. Kuhl et al., 1992; Werker and Tees, 2002)^5^. Incidentally, it has to be noted that recent studies have shown that the characteristic “hyperarticulation” of infant-directed speech may be restricted to these suprasegmental levels of prosody, given that rather than hyperarticulated, phonemic contrasts can be hypoarticulated in infant-directed speech, i.e., that mothers hyperarticulate their infant-directed speech in prosodic aspects, but in segmental aspects mothers may “speak less clearly to infants than to adults” (cf. Martin et al., 2014).

Now, several authors have proposed that the early acquired rhythmic properties of languages are not idiosyncratic and isolated properties, but rather that they are strongly correlated with the particular syntactic properties of the particular languages (i.e., that there are correlations between rhythmic patterns and syntactic patterns in that languages tend to cluster with the same rhythmic and syntactic properties, conforming linguistic typologies). Furthermore, the explanation of this typological clustering is proposed to derive from the fact that rhythmic patterns serve to bootstrap or catalyze the acquisition of the specific syntactic patterns of each language (cf. *i.a*. Mehler et al., 1988; Christophe et al., 2003; Bernard and Gervain, 2012; Gervain and Werker, 2013; Langus and Nespor, 2013)^6^. In particular, a number of authors have proposed that the relative order between heads and their complements strongly correlates with the rhythmic type of the language. A number of experiments have shown that languages whose correlates of phrasal accent are increases in duration and intensity tend to be head-initial (with a Verb-Object word order) whereas languages that realize stress through a combination of higher pitch and intensity (and possibly also duration) tend to be head-final (with an Object-Verb word order)^7^. This generalization is known as the ‘iambic-trochaic law’ (cf. *i.a*. Hayes, 1995; Nespor et al., 2008; Shukla and Nespor, 2010), which is taken to be a basic law of grouping based on general auditory perception (i.e., not specific to language) that states that units (language or music) that differ in intensity tend to be grouped as constituents in which the most prominent element comes first, and units that differ in duration are grouped as constituents in which the most prominent element comes last^8^. As Nespor et al. (2008) put it, “if [their] proposal is on the right track, one of the basic properties of syntax can be learned through a general mechanism of perception.”

This line of reasoning is reinforced by recent studies such as Gordon et al. (2015) suggesting that there is a correlation between rhythm perception skills and morpho-syntactic production in children with typical language development (and note also that a strong association between reading skills and meter perception and rhythm processing has been found; Flaugnacco et al., 2014; Leong and Goswami, 2014). Likewise, studies like Zumbansen et al. (2014), Leong and Goswami (2014) report the beneficial effects of both pitch and rhythm in the clinical therapy for patients with Broca's aphasia.

In the next section, I argue for the natural combination of the “globularization” and “prosodic bootstrapping” hypotheses.

## 4. Synthesis: the “globularization hypothesis” as a developmental frame for the “prosodic bootstrapping” hypothesis

Let us focus on the two main ideas that we have seen so far, which are that (i) according to the “globularization hypothesis” of Boeckx and Benítez-Burraco (2014a,b), the postnatal globularization of the brain is an essential ingredient for the development of our syntactic capacities, and that (ii) according to the “prosodic bootstrapping hypothesis” of Mehler et al. (1988), Christophe et al. (2003), Bernard and Gervain (2012), Gervain and Werker (2013), Langus and Nespor (2013) and others, children use prosody in order to infer the syntactic pattern of the language they are acquiring.

The combination of these two hypotheses brings about an interesting picture regarding language acquisition: it leaves room for a delay in the acquisition of syntax with respect to prosody. If the “globularization hypothesis” is correct, syntactic capacities develop some months after birth and if the “prosodic bootstrapping hypothesis” is correct, children use prosody as a guiding principle for acquiring syntax. That is, babies may have a rich knowledge of prosody (as pure melodic patterns, unrelated to any syntactic structure) by the moment they develop the capacity to start parsing syntax. Crucially, all the data discussed in Sections 2 and 3 point in that direction: after some months of pre- and post-natal experience with linguistic input, babies have a fairly good knowledge of the prosodic properties of the language(s) spoken around them, this knowledge being arguably well established by the time they develop the structures necessary for parsing syntax. Therefore, babies will be able to use all this phonological knowledge as a guiding principle to discover the syntax behind the acoustic signals. As a matter of fact, the hypothesis by Boeckx and Benítez-Burraco (2014a,b) can provide a developmental rationale for the prosodic bootstrapping hypothesis of early language acquisition. Given Boeckx and Benítez-Burraco's hypothesis, it is natural for a rich phonological knowledge to be established *before* the syntactic ability develops, for the necessary mechanisms for phonological acquisition are present at birth. Then, endowed with a rich prosodic knowledge, language-acquiring children will be able to use it as a bias for hypothesizing the syntactic pattern of the target language (which in a Bayesian model could take the form of an informed prior). In an nutshell, the prosodic bootstrapping hypothesis claims that beyond the observed typological correlation between prosodic and syntactic patterns, there is a causal developmental connection between them: babies use prosody to guess the syntactic pattern of their target language and my proposal is that the globularization hypothesis provides a natural developmental frame for the prosodic bootstrapping hypothesis, for it presents a relatively late syntactic development *vis à vis* the prosodic development.

As a last remark, it should be noted that the globularization hypothesis—besides capturing the fact that prosodic knowledge precedes syntactic knowledge—also leaves room for explaining why first language acquisition is fast, but not immediate, for not all the necessary neurocognitive machinery would be established from birth (cf. Boeckx and Benítez-Burraco, 2014a,b). Even if innately specified, some maturation is in order for a fully language-ready brain.

## Funding

This work was partially funded by the following agencies: the European Union's Seventh Framework Program (AThEME 613465), the Basque Government (IT769-13), and the Spanish MINECO (FFI2012-38064-C02-01, FFI2014-53675-P, FFI2013-41509-P).

### Conflict of interest statement

The author declares that the research was conducted in the absence of any commercial or financial relationships that could be construed as a potential conflict of interest. The reviewer, Silvia Martínez Ferreiro, and handling editor, Cedric Boeckx, declared their shared affiliation, and the handling editor states that the process nevertheless met the standards of a fair and objective review.

## Footnotes

^1^However, infants do not seem to show preferences for their father's voice (cf. Ward and Cooper, 1999).

^2^See Telkemeyer et al. (2009) for a near-infrared spectroscopy and EEG study showing that a right hemispheric lateralization for slow acoustic modulations (characteristic of prosodic features) is present at birth (see also Telkemeyer et al., 2011).

^3^See also Wang et al. (2015) for a proposal about the brain areas in charge of the human-specific ability for the integration of multiple features in abstract pattern learning.

^4^A reviewer rightly notes that these studies are performed with adult subjects, not with babies. Indeed, we need infant studies to assess the development of rhythmic brain oscillations (see Musacchia et al., 2015, for some of the first reported evoked oscillations analyses in infants).

^5^In fact, lack of adequate acquisition of the phonology of the target language can generate disorders such as dyslexia (Paulescu et al., 2001; Goswami, 2011; Saralegui et al., 2014; see also Benítez-Burraco, 2013)

^6^Donegan and Stampe (1983, 2004) have even proposed a “holistic typology” based on rhythmic grounds in order to account for the polarized structural divergence of languages like Munda and Mon Khmer.

^7^In turn, speakers of languages with different rhythmic patterns like English vs. Japanese tend to behave differently in the way in which they group nonlinguistic stimuli (Iversen et al., 2008).

^8^In a recent study de la Mora et al. (2013) observed that rats group sequences based on pitch variations as trochees, but that they do not group sequences varying in duration as iambs.
